# Einfluss der Arbeitsbedingungen und des Gehalts auf die Leiharbeit für Intermediate-Care- und Intensivstationen: Teilergebnisse einer bundesweiten Umfrage

**DOI:** 10.1007/s00063-022-00929-1

**Published:** 2022-06-10

**Authors:** C. Hermes, K. Blanck-Köster, U. Gaidys, E. Rost, C. Petersen-Ewert

**Affiliations:** 1 Hochschule für Angewandte Wissenschaften Hamburg (HAW Hamburg), Alexanderstrasse 1, 20099 Hamburg, Deutschland; 2 Fakultät Wirtschaft & Soziales – Department Pflege & Management, Hochschule für angewandte Wissenschaften Hamburg, Hamburg, Deutschland; 3 IU Internationale Hochschule GmbH, IU University of Applied Sciences, Juri-Gagarin-Ring 152, 99084 Erfurt, Deutschland

**Keywords:** Arbeitnehmerüberlassung, Festangestellte, Zeitarbeit, Qualität der Gesundheitsversorgung, Intensivpflege, Agency worker, Permanent employees, Temporary work, Quality of health care, Critical care

## Abstract

**Hintergrund:**

Leiharbeit in der Pflege wird als Arbeitsform verwendet, um offenen Personalstellen in Kliniken zu begegnen. Sowohl Krankenhausträger als auch Pflegekräfte sehen dies aus unterschiedlichen Gründen kritisch.

**Ziel:**

Zweck dieser Untersuchung war es herauszufinden, welches persönliche Nettoeinkommen Pflegekräfte von deutschen Intensivstationen und Intermediate-Care-Stationen als „gerecht und ausreichend“ für ihre Tätigkeit empfinden und welchen Einfluss das Gehalt auf die Wechselwilligkeit in die Leih‑/Zeitarbeit bzw. wieder zurück in eine Festanstellung haben.

**Methode:**

Von September bis Oktober 2020 wurde eine anonymisierte Onlineumfrage unter Pflegenden von Intermediate-Care-Stationen, Intensivstationen und Funktionsbereichen im deutschsprachigen Raum durchgeführt. Die Auswertung erfolgte mittels deskriptiver Statistik.

**Ergebnis:**

Von 1203 Teilnehmer_innen (TN) konnten 1036 (86 %) in Deutschland Arbeitende ausgewertet werden. Die Frage nach dem persönlichen Nettoeinkommen wurde von 1032 (99 %) TN beantwortet. Der überwiegende Anteil der Befragten (*n* = 522) gibt an, über ein persönliches Nettoeinkommen von 2000–2999 €/Monat zu verfügen. Der Wunsch, in die Leiharbeit zu gehen, ist in der untersuchten Stichprobe geringer, je höher das persönliche Nettoeinkommen ist. Die TN in Festanstellung ohne Nebenerwerb empfinden ein persönliches Nettoeinkommen von 3200 €/Monat (Median 3200 €; IQR 2800–3800 €) als ausreichend und gerecht für ihre Tätigkeit. Von den Leiharbeiter_innen gaben 142 Personen an, dass ein persönliches Nettoeinkommen von 3200 €/Monat (Median 3200 €; Interquartilsabstand 3000–3950 €) ausreichend wäre, um von der Leiharbeit zurück in eine Festanstellung zu wechseln.

**Schlussfolgerung:**

Die Intensivpflegenden dieser Umfrage empfinden ein Gehalt von 3200 €/Monat netto als ausreichend und gerecht für ihre Tätigkeit. Die Gehaltshöhe kann ein Parameter für die Entscheidung sein, in die Leiharbeit zu gehen, aber auch um wieder in die Festanstellung zu wechseln. Unabhängig vom Gehalt wurden bessere Arbeitsbedingungen für alle befragten Gruppen als essenzieller Bestandteil in Bezug auf die Arbeitszufriedenheit angegeben.

## Abstract (en)

**Background:**

Agency nursing is used as a form of labour to counter vacant staff positions in hospitals. Stakeholders and nurses might view this critically for different reasons.

**Aim:**

The aim of this study was to evaluate what individual net income nurses in German intensive care units and intermediate care units consider “fair and acceptable” for their work. Furthermore, what influence does salary have on the willingness to change to agency nursing or back to a permanent position.

**Methods:**

From September–October 2020, an anonymous online survey was performed among nurses of intermediate care units, intensive care units and special care units in German-speaking countries. The results were evaluated using descriptive statistics.

**Result:**

Of 1203 participants, 1036 (86%) of those working in Germany could be evaluated. The question about the individual net income was answered by 1032 (99%) participants. The majority of respondents (*n* = 522) stated that they had an individual net income of 2000–2999 €/month. The higher the level of the net income, the lower the willingness to switch to agency work. The participants in permanent employment only perceive a net income of 3200 €/month (median 3200 €; interquartile range [IQR] 2800–3800 €) as acceptable and fair for their work. In all, 142 agency nurses stated that an individual net income of 3200 €/month (median 3200 €; IQR 3000–3950 €) would be sufficient to move from agency nursing back into permanent employment.

**Conclusion:**

The intensive care nurses in this survey consider a salary of 3200 €/month as acceptable and fair for their work. The salary level can be a parameter for the decision to go into agency work, but also to move back to permanent employment. Regardless of the salary, better working conditions were indicated as an essential element in terms of job satisfaction for all respondent groups.

## Einleitung

In deutschen Kliniken sind in den letzten 30 Jahren vor der SARS-CoV-2-Pandemie die Behandlungsfälle um 25 % gestiegen, während gleichzeitig die Anzahl der Krankenhäuser von 2400 auf 1942 gesunken ist [18]. Hieraus resultiert eine deutliche Arbeitsverdichtung, die durch den demographischen Wandel in den nächsten Jahren aufgrund eines weiteren Rückgangs der Bevölkerung im Erwerbsalter und eines nominellen Anstiegs von Senioren und Pflegeempfängern verstärkt wird [23]. Zunehmender ökonomischer Druck im Gesundheitssystem führte in den letzten Jahren zu einer massiven Personaleinsparung v. a. im pflegerischen Bereich [19]. Dies steht im Gegensatz zum eigentlichen Versorgungsbedarf [13].

Obwohl es in Deutschland, verglichen mit allen anderen europäischen Ländern, überdurchschnittlich viele Intensiv- und Intermediate-Care-Betten bezogen auf die Einwohnerzahl gibt (etwa 30 Betten pro 100.000 Einwohner_innen; [20]), kommt es paradoxerweise in diesem Bereich immer wieder zu Engpässen in der Patient_innenversorgung. Diese Engpässe in der intensivmedizinischen Versorgung sind maßgeblich darauf zurückzuführen, dass sowohl die Anzahl der Intensivbetten als auch die Behandlungsfälle in Deutschland kontinuierlich gestiegen sind, während im selben Zeitraum die Anzahl der Pflege- und Fachpflegekräfte abgenommen hat [12]. Daraus folgend können Betten und Behandlungsplätze in der Regel- und Notfallversorgung nicht belegt bzw. müssen gesperrt werden. Eine messbare Konsequenz ist, dass mittlerweile die Anzahl der Pflegekräfte auf deutschen Intensivstationen nicht mehr ausreichend ist, um eine kontinuierliche und qualitativ hochwertige Patient_innenversorgung sicherzustellen [14]. In vielen deutschen Kliniken wird versucht, dem akuten Personalmangel durch unterschiedliche Maßnahmen, wie z. B. durch flexible Arbeitszeitmodelle und den Einsatz von Leiharbeiter_innen, entgegenzuwirken, um die Patient_innenversorgung aufrechtzuhalten [4, 16].

Allerdings führen in Deutschland vor allem die quantitativen Anforderungen an Pflegende zu einem zunehmenden Burn-out, steigenden Fehlzeiten und zu der vermehrten Absicht, das Berufsfeld in Gänze zu verlassen [22]. Zudem haben Krankenhausmanager_innen zunehmend Schwierigkeiten, Pflegepersonal zu rekrutieren und/oder zu halten. Für die 1990er-Jahre wird beschrieben, dass die Leiharbeit zu einer Stabilisierung der Arbeitsmärkte und Löhne geführt hat [4, 11]. Mittlerweile wird an dieser Arbeitssituation zunehmend Kritik laut. Es werden negative Auswirkungen auf die Versorgungsqualität durch Leiharbeiter_innen berichtet. Demnach würden sie in medizinischen Notfällen nicht richtungsweisend vorgehen oder seien oft wenig vertraut mit Hygieneplänen, IT-Systemen sowie Evakuierungs- und Alarmierungsplänen für Großschadenslagen usw. Zudem sei eine Bindung und Vertrautheit der Leiharbeitnehmer_innen zum Pflegebedürftigen mit negativen Auswirkungen (z. B. auf Demenzpatienten) nicht gegeben und stünde im Widerspruch zu einer notwendigen Bezugspflege. Die Kritik führt dazu, dass ein Verbot dieser Arbeitsform diskutiert wurde [3]. Als Begründung für ein Verbot wird vorgebracht, dass höhere Gehälter bei vermeintlich besseren Arbeitszeiten (weniger Stunden und gezielte Auswahl von Arbeitsschichten) für Leiharbeitnehmer_innen vorliegen und Stammpersonal systematisch mit diesen Argumenten abgeworben wird. Das würde die Arbeitsbedingungen der verbliebenen Personen in Festanstellung deutlich verschlechtern und zu einer erhöhten Belastung führen [3, 5, 17]. Bei der Darstellung wird nicht zwischen den einzelnen Sektoren in der Pflege und insbesondere nicht zwischen der Fach- und Funktionspflege, wie der Intensivpflege und der sog. Normalstationspflege, unterschieden [1]. Dies ist insofern wichtig, da diese Bereiche nicht nur eine höhere Spezialisierung aufweisen, sondern auch einen anderen Betreuungsschlüssel haben. Zudem konnte in einem systematischen Review kein Zusammenhang zwischen einem veränderten Patient_innenoutcome und dem Einsatz von Leiharbeiter_innen auf der Intensivstation identifiziert werden. In der Bewertung von Outcomeparametern scheint es auch eine Diskrepanz zwischen subjektiven Wahrnehmungen und messbaren Ergebnissen zu geben [10].

In der vorliegenden Studie wird der Begriff Leiharbeiter_innen für alle Formen der Leih‑/Zeitarbeit und Arbeitnehmerüberlassung verwendet. Eine reine freiberufliche und/oder selbständige Tätigkeit als Pflegekraft ist in deutschen Kliniken durch das aktuelle Urteil des Bundessozialgerichtes (Az. BSG B 12 R 11/18 R) nicht mehr möglich.

## Zielsetzung

Das Ziel dieser Untersuchung ist es herauszufinden, welches persönliches Nettoeinkommen (pNEK) Pflegekräfte von deutschen Intensivstationen und Intermediate-Care-Stationen als „gerecht und ausreichend“ für ihre Tätigkeit empfinden und welchen Einfluss dieses Gehalt auf die Wechselwilligkeit in die Leih‑/Zeitarbeit bzw. wieder zurück in eine Festanstellung hat.

## Methode

Vom 05. September 2020 bis zum 06. Oktober 2020 wurde eine anonymisierte offene Onlineumfrage in einem quantitativen Design mittels der Onlineplattform SurveyMonkey (https://www.surveymonkey.de/) unter Pflegenden von Intensivstationen und anderen Fach- und Funktionsbereichen im deutschsprachigen Raum durchgeführt. Aufgrund des Umstands, dass fast alle auswertbaren Antworten aus Deutschland kamen, wurde im Verlauf die Auswertung auf diesen Raum begrenzt. Die gesamte Umfrage wurde in Übereinstimmung mit den Empfehlungen zur Durchführung von Onlineumfragen erstellt [9]. Die Befragung stellt ein Querschnittdesign mit einer Ex-post-facto-Analyse dar.

### Stichprobe

Die Grundgesamtheit der Pflegefachpersonen auf Intensivstationen und Überwachungsbereichen ist in Deutschland statistisch nicht belastbar erfasst. Bei dieser Befragung ist am ehesten von einer uneingeschränkten Zufallsstichprobe auszugehen [15]. Durch das Design und den Zeitraum der Befragung sind alle Ergebnisse als Tendenz zu betrachten [21].

### Durchführung

Der Teilnahmeaufruf wurde in 3 bundesweit verfügbaren Fachzeitschriften („*PflegenIntensiv“, „Intensiv“*, „*Medizinische Klink – Intensiv und Notfallmedizin*“) und deren Onlineportalen veröffentlicht. Zusätzlich wurde die Umfrage über Berufsverbände und deren E‑Mail-Verteiler sowie deren Kanäle der sozialen Medien (Twitter, Facebook, WhatsApp) bekanntgemacht, verteilt und teilweise auf deren Homepage eingebunden. Beteiligt waren die Deutsche Gesellschaft für internistische Intensiv- und Notfallmedizin e. V. (DGIIN), die Deutsche Gesellschaft für Fachkrankenpflege und Funktionsdienste e. V., die Schweizerische Gesellschaft für Intensivmedizin und die Österreichische Gesellschaft für Allgemeine Intensivmedizin und Notfallmedizin. Ferner wurden Kliniken in den deutschsprachigen Gebieten von Belgien, Luxemburg, Lichtenstein und Italien (Bozen) persönlich angeschrieben. Die Einladungen enthielten eine Kurzbeschreibung der Umfrage sowie einen Link zur zugehörigen Website und den Aufruf, die Einladung an andere mögliche TL in einem Schneeballsystem weiterzuleiten, sodass eine Berechnung der tatsächlichen Rücklaufquote nicht möglich war. Darüber hinaus wurden die Einladung und der Link auf diversen Fachtagungen und Schulungen persönlich und durch Kolleginnen und Kollegen direkt beworben.

Der Onlinefragebogen wurde nach Beratungen mit Psychologen für Eignungsdiagnostik erstellt. Die TN konnten jederzeit das Ausfüllen des Fragebogens unterbrechen oder auch abbrechen. Die Teilnahme war freiwillig. Es wurden keine Entschädigungen, sonstige Vergünstigungen, Gewinne oder Preise für eine Teilnahme offeriert. Rückschlüsse auf einzelne Personen oder Kliniken sind theoretisch in bestimmten Konstellationen denkbar, z. B. durch besondere soziodemographische Daten im Zusammenhang mit anderen Einzelmerkmalen oder durch die IP-Adresse (Internetprotokoll). Die IP-Adresse wurde konform mit der Datenschutzgrundverordnung erfasst. Sie wurde nur temporär zwischengespeichert und mit bisherigen Teilnahmen an dieser Umfrage verglichen. Damit sollte a) eine Unterbrechung und nahtloses Fortführen der Umfrage am selben Endgerät möglich sein und b) verhindert werden, dass vom selben Endgerät aus mehreren Fragebögen ausgefüllt werden. Die Daten wurden daraufhin vom Erstautor und einem Statistiker gesichtet. Diesbezüglich wurden keine Auffälligkeiten und Einzelmerkmale gefunden, die einen Rückschluss auf einzelne Personen oder Kliniken zulassen würden. Auf einen Ethikantrag wurde nach der Einschätzung eines Mitglieds des Ethikkomitees der evangelischen Kliniken Bielefeld und gemäß der Einschätzung der betreuenden Professorin an der Hochschule für angewandte Wissenschaften in Hamburg verzichtet, da die Teilnahme freiwillig war und keine vulnerable und/oder vom Forscher_innenteam abhängige Gruppe befragt wurde. Erinnerungen zur Teilnahme wurden über die E‑Mail-Verteiler und soziale Medien gestreut. Die Umfrage wurde geschlossen, als an mehreren Tagen hintereinander weniger als 5 Antworten in der Reportanalyse verzeichnet wurden.

### Messinstrument

Als Grundlage für die Konzeption der Fragestellungen diente eine zuvor durchgeführte und Onlineumfrage der Landespflegekammer Niedersachen, die das Deutsche Institut für angewandte Pflegeforschung e. V. durchgeführt hatte. Der Fragebogen wurde vorab einem Pretest zur Plausibilitätsprüfung unterzogen. Der Pretest wurde unter insgesamt 40 Festangestellten mit und ohne Nebenerwerb und unter Leiharbeiter_innen, die in der Intensivpflege tätig sind, zu gleichen Anteilen durchgeführt. Darunter waren sowohl Absolventen als auch noch in Weiterbildung (Anästhesie und Intensivpflege) befindliche Personen und akademisierte Pflegende. Die Ergebnisse wurden nicht in die Analyse einbezogen, allerdings im Vorfeld u. a. zur Präzisierung der Fragestellungen und semantischen Anpassung verwendet.

Eine wichtige Anpassung war u. a., nur nach dem Nettoeinkommen zu fragen, da die meisten Teilnehmer_innen dies eher angeben konnten als ihr tatsächliches Bruttogehalt. Der endgültige Fragebogen bestand aus mehreren eigenständigen und teilweise aufeinander aufbauenden Teilen mit insgesamt 84 Fragen mit vereinzelten Freitextantworten (vollständiger Fragebogen kann beim Erstautor angefordert werden). Der Fragebogen wies elektronische Verschachtelungen auf, die auf logischen Weiterleitungen unter Berücksichtigung der getroffenen Antworten beruhten. So war gewährleistet, dass Leiharbeiter_innen und Festangestellte ohne Nebenerwerb sowie Festangestellte mit einem Nebenerwerb in oder außerhalb der Leiharbeit für sie spezifische und teilweise unterschiedliche Fragen beantworten mussten. Ebenso wurde bei allen Festangestellten unterschieden, ob diese bereits mit Leiharbeiter_innen beruflich zusammengearbeitet haben oder nicht. Die TN haben die Möglichkeit, bei der Beantwortung der Fragen hin- und herzuspringen, hatten aber keinen Zugang zu den Gesamtergebnissen. Um Fragereiheneffekte zu vermeiden [6], wurden ebenfalls automatisierte Randomisierungen für die Fragen verwendet. Als Frage- und Antworttypen wurden dichotome Fragen und Antworten, Mehrfachauswahl und Mehrfachantworten, Freitextantworten und auch Fragen mit einer Bewertungsskala von 0–100 verwendet. Als Skalen wurden modifizierte Likert-Skalen und vereinzelt Skalen als semantisches Differenzial verwendet.

### Auswertung

Die Datenauswertung erfolgte über das statistische Datenverarbeitungsprogramm JASP‑0,14® in einer Open-Source-Lizenz. Eine Operationalisierung und Kategorisierung der offenen Antworten erfolgten durch Zuordnung zu Kategorien, sofern dies eindeutig möglich war, und/oder unter sonstige. Freitextformulierungen wurden dabei in der Regel für ergänzende Kommentare genutzt. Für manche Fragen wurden als Antwortmöglichkeit eine den im Internet gebräuchlichen Bewertungen nachempfundene 5‑stufigen Sternskala verwendet. Die Kategorie „ich weiß nicht“ wurde bewusst nicht verwendet, damit TN eine Entscheidung treffen mussten. Um einen Selektionsbias aufgrund von Voreingenommenheit durch den Untersucher zu vermeiden, wurden Kategorisierungen und Operationalisierung der Variablen zuvor durch einen in der empirischen Sozialforschung erfahrenen an dieser Befragung nicht beteiligten unabhängigen Dritten vorgenommen.

Die Auswertung der univariaten und bivariaten Messzahlen erfolgt mittels deskriptiver Statistik. Es werden das arithmetische Mittel (MW) und die Standardabweichung (SD) für normalverteilte Daten und der Median (MD) mit Interquartilsabstand (IQR) für nicht normalverteilte Variablen angegeben. Ferner wird die Häufigkeit als Absolutwert und in Prozent (%) dargestellt. Die Signifikanzberechnung erfolgte mittels χ^2^-Tests bei 2 und bei mehr als 2 Gruppen mittels Varianzanalyse (ANOVA). Für die ANOVA wurde eine Post-hoc-Analyse mit Scheffé-Anpassung durchgeführt. Wenn die Voraussetzungen für ein parametrisches Verfahren nicht gegeben waren, wurde stattdessen der Kruskal-Wallis-Test durchgeführt. Zusammenhangsprüfungen der intervallskalierten Variablen werden über die Regressionsanalyse durchgeführt.

## Ergebnisse

### Allgemeiner Teil und soziodemographische Ergebnisse

Die im Folgenden dargestellten Ergebnisse beziehen sich auf beendete Fragen zum Einkommen von den Teilnehmer_innen aus Deutschland, die im Zeitraum vom 05. September 2020 bis zum 06. Oktober 2020 durchgeführt wurde. Die Darstellung der weiteren Ergebnisse folgt in einer gesonderten Veröffentlichung. Insgesamt haben 1203 Personen am Onlinefragebogen teilgenommen. Die allgemeinen Pflichtangaben beantworteten 1036 (86 %) TL vollständig. Diese Angaben wurden im Folgenden analysiert: Der überwiegende Anteil der Teilnehmer_innen war weiblich mit Hauptwohnsitz in Deutschland und einer Klinik als Hauptarbeitgeber (Tab. 1).

**Table 1:** 

Variable	Ausprägung	*n*	%
Geschlecht	Weiblich	619	59,8
	Männlich	413	39,8
	Divers	4	0,4
Hauptwohnsitz	Deutschland	998	96,2
	Anderer Ort	39	3,8
Bundesland	Baden-Württemberg	76	7,8
	Bayern	89	9,2
	Berlin	34	3,5
	Brandenburg	7	0,7
	Bremen	7	0,7
	Hamburg	76	7,8
	Hessen	66	6,8
	Mecklenburg-Vorpommern	8	0,8
	Niedersachsen	76	7,8
	Nordrhein-Westfalen	368	37,9
	Rheinland-Pfalz	84	8,6
	Saarland	9	0,9
	Sachsen	29	2,9
	Sachsen-Anhalt	4	0,4
	Schleswig-Holstein	30	3,1
	Thüringen	9	0,9
Weiterbildung	Fachweiterbildung	591	65,4
	Praxisanleiter	245	27,1
	Sonstiges	68	7,5
Akademisierung	Bachelor-Studium	114	11
	Master-Studium	57	6

### Tätigkeit- und Einkommensverteilung

**Figure 1:**
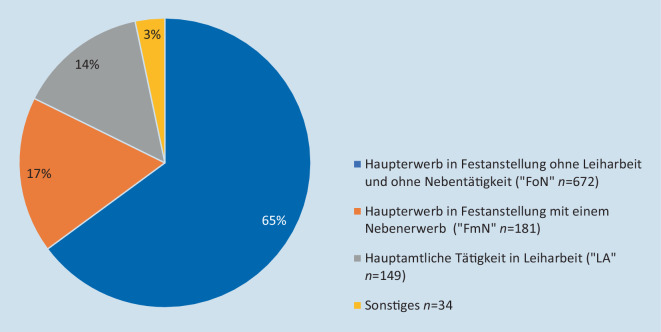

**Figure 2:**
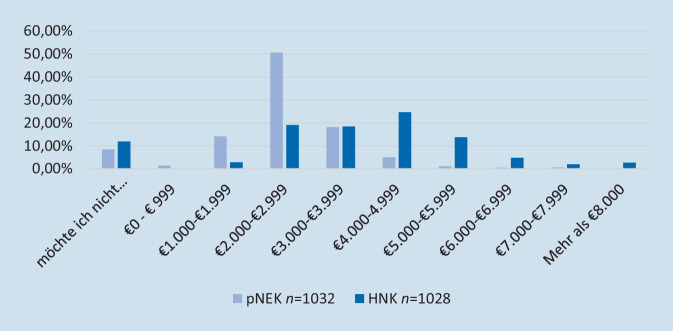

Von den 1036 analysierten Teilnehmer_innen gaben *n* = 672 TN (65 %) an, einen Haupterwerb in Festanstellung ohne Leiharbeit und ohne Nebentätigkeit auszuüben. Weitere *n* = 181 TN (17 %) gaben an, einem Haupterwerb in Festanstellung mit einem Nebenerwerb nachzugehen. Ein Anteil von *n* = 50 TN (5 %) hat hierfür neben der Festanstellung einen Nebenerwerb in der Leiharbeit, *n* = 131 TL (13 %) gaben an, einem Nebenerwerb ohne Leiharbeit nachzugehen. Eine hauptberufliche Tätigkeit als Leiharbeiter_in z. B. über eine Vermittlungsfirma oder andere Agentur gaben *n* = 149 TN (14 %) an. Eine sonstige Tätigkeit gaben *n* = 34 (3 %) der TN an (Abb. 1). Die Frage nach dem persönlichen Nettoeinkommen (pNEK) wurde von *n* = 1032 TN (99 %) beantwortet, wobei *n* = 87 (8 %) dieses nicht beziffern wollten. Der überwiegende Anteil der TN (*n* = 522, 50 %) gibt an, über ein pNEK von 2000–2999 € zu verfügen. Die Frage zum Haushaltsnettoeinkommen (HNE) wurde von *n* = 1028 TN (99 %) beantwortet, wobei *n* = 122 (12 %) dieses nicht beziffern wollten. Ein HNE von 4000–4999 € gaben *n* = 253 TN (24 %) an, weitere *n* = 196 TL (19 %) gaben 2000–2999 €, *n* = 189 TL (18 %) 3000–3999 € an. Bei *n* = 297 TN (29 %) entspricht das pNEK dem HNE (Abb. 2). Es besteht ein signifikanter bivariater Zusammenhang zwischen dem persönlichen Nettoeinkommen und dem Tätigkeitsumfang (R^2^ = 52 %, t [564] = 13,568; *p* < 0,001, β = 0,499) und kein bivariater Zusammenhang zwischen dem Geschlecht (R^2^ = 0,90 %, t [564] = −2,633; *p* = 0,009, β = −0,097). Insgesamt zeigt sich das durchgeführte multiple Regressionsmodell mit den Variablen Geschlecht und dem Tätigkeitsumfang als signifikant (R^2^ = 0,53, F [1, 563] = 6,933, *p* = 0,009), wobei auch hier nur der Tätigkeitsumfang selbst einen signifikanten Zusammenhang mit dem persönlichen Nettoeinkommen aufzeigt.

Die Fragestellung nach den Unterschieden in der Gehaltsverteilung der einzelnen untersuchten Gruppen konnte mittels Varianzanalyse analysiert werden. Die einzelnen Gehaltsangaben ergeben gemittelt eine klare Verteilung: Leiharbeiter_innen verdienen mehr als Festangestellte (t = −7,192; *p* < 0,001; d = −0,687) und mehr als Festangestellte mit einem Nebenerwerb außerhalb der Leiharbeit (t = −5,687; *p* < 0,001; d = −0,776). Für keine der anderen untersuchten Gruppen ergeben sich weitere signifikante Unterschiede (alle *p* > 0,050). Beim Nebenerwerb zeigt sich die nichtsignifikante Tendenz (*p* < 0,100), dass Festangestellte mit einem Nebenerwerb in der Leiharbeit ebenfalls finanziell bessergestellt sind als Festangestellte mit einem Nebenerwerb außerhalb der Leiharbeit. Dieser deskriptiv ersichtliche Effektunterschied war jedoch inferenzstatistisch nicht signifikant. Tendenziell zeigt sich, dass der Wunsch, in die Leiharbeit zu gehen, in der untersuchten Stichprobe geringer ist, je höher das persönliche Nettoeinkommen ist. Dieser Eindruck konnte jedoch nicht inferenzstatistisch bestätigt werden. Je höher das pNEK ist, desto geringer ist der Wunsch, in die Leiharbeit zu gehen. Dieser Effekt ist jedoch nicht signifikant.

Die Fragestellung „Verdienen Leiharbeiter_innen mehr als Festangestellte?“ wurde mittels einer einfaktoriellen Varianzanalyse überprüft: Hierbei wurde das pNEK der Teilnehmer_innen auf Unterschiede in den Gruppen Leiharbeitnehmer_innen, Festangestellte ohne Nebenerwerb, Festangestellte mit einem Nebenerwerb, dabei in der Unterscheidung Festangestellte mit einem Nebenerwerb in der Leiharbeit und Festangestellte mit einem Nebenerwerb außerhalb der Leiharbeit, untersucht. Innerhalb der ersten deskriptiven Untersuchung (Tab. 2) lässt sich feststellen, dass die befragten Leiharbeitnehmer_innen mit einem MW = 3248,15 € (SD = 1012,74 €) das höchste Nettoeinkommen aller befragten Personen aufwiesen, während Festangestellte im MW = 2568,97 € mit SD = 681,74 € (Festanstellung mit Nebenerwerb ohne Leiharbeit) und im MW = 2797,87 € mit SD = 1121,24 € (Festanstellung mit Nebenerwerb in Leiharbeit) verdienen. Mit H (4) = 61.262 und *p* < 0,001 kann der aufgefundene Haupteffekt der Varianzanalyse bestätigt werden.

**Table 2:** 

Gruppe	MW	SD	SE	*N*
Festanstellung, Nebenerwerb in Leiharbeit	2797 €	1121 €	137 €	47
Festanstellung, Nebenerwerb ohne Leiharbeit	2568 €	681 €	87 €	116
Festanstellung	2603 €	921 €	37 €	618
Leiharbeit	3248 €	1012 €	81 €	135
Sonstige	3000 €	1615 €	192 €	24

^a^Gehalt wurde als Spanne abgefragt und nicht ins Verhältnis zum Stellenumfang gesetzt, wodurch die hohe SD erklärt wird

In der anschließend durchgeführten Post-hoc-Analyse (Tab. 3) zur Feststellung der Unterschiede innerhalb der Untersuchungsgruppen wurde die Scheffé-Korrektur für paarweise Gruppenvergleiche angewandt. Hier zeigen sich zwischen den Gruppen Leiharbeit und Festanstellung mit *p* < 0,001 und d = 0,687 sowie Leiharbeit und Festanstellung mit Nebenerwerb ohne Leiharbeit mit *p* < 0,001 und d = 0,776 signifikante mittelstarke Gruppenunterschiede. Keine der anderen Gruppenvergleiche wird in dieser Analyse signifikant. Beim Nebenerwerb zeigt sich die Tendenz, dass Festangestellte mit einem Nebenerwerb in der Leiharbeit ebenfalls finanziell bessergestellt sind als Festangestellte mit einem Nebenerwerb außerhalb der Leiharbeit. Dieser Effekt war jedoch nicht signifikant. Je höher das pNEK ist, desto geringer ist der Wunsch, in die Leiharbeit zu gehen. Dieser Effekt ist jedoch nicht signifikant.

**Table 3:** 

Gruppe	Variable	∆ MW	SD	t	Cohen’s d	*p* Scheffé*
Festanstellung (FA)	FAmNL	−194,312	142,74	−1,361	−0,208	0,763
	FmNoL	34,594	95,456	0,362	0,039	0,998
	LA	−644,588	89,622	−7,192	−0,687	< 0,001***
	Sonstige	−396,44	196,267	−2,02	−0,415	0,396
Festanstellung, Nebenerwerb in Leiharbeit (FmNL)	FmNoL	228,907	163,115	1,403	0,275	0,741
	LA	−450,276	159,771	−2,818	−0,432	0,095
	Sonstige	−202,128	236,675	−0,854	−0,155	0,948
Festanstellung, Nebenerwerb ohne Leiharbeit (FmNoL)	LA	−679,183	119,432	−5,687	−0,776	< 0,001***
	Sonstige	−431,034	211,548	−2,038	−0,475	0,387
Leiharbeit [LA]	Sonstige	248,148	208,98	1,187	0,221	0,842

*„p*-value adjusted for comparing a family of 5 – Cohen’s d does not correct for multiple comparisons“

*p < 0,05; *** *p* < 0,001

**Figure 3:**
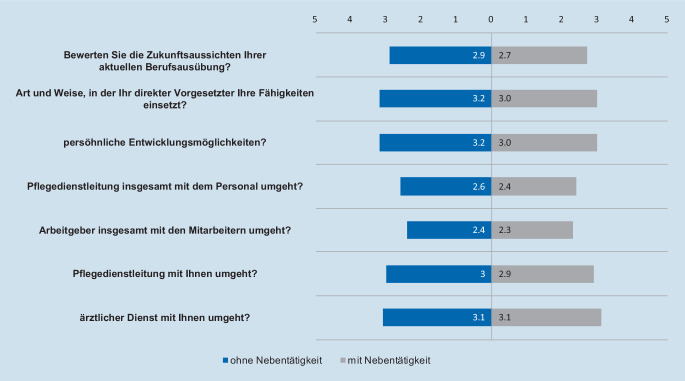

Neben der Angabe des tatsächlichen persönlichen Nettoeinkommens wurden die TN auch gebeten anzugeben, welches Gehalt sie als ausreichend und gerecht empfinden. Diese Frage diente als indirekter Indikator für eine mögliche Unzufriedenheit. Dabei gaben 607 TN in Festanstellung ohne Leiharbeit ein pNEK im Median von 3200 €/Monat als ausreichend und gerecht an (Median 3200 €; IQR 2800–3800 €). Die TN in Festanstellung mit einem Nebenerwerb (*n* = 166; 16 %) empfanden im Median ein pNEK von 3000 €/Monat (Median 3000 €; IQR = 3000–3500 €) als ausreichend und gerecht. Von den Leiharbeiter_innen gaben 142 Personen an, dass ein pNEK von 3200 €/Monat (Median 3200 €; IQR 3000–3950 €) ausreichend wäre, um aus der Leiharbeit auszutreten. Die Fragen zur Zufriedenheit mit verschiedenen Aspekten der aktuellen Tätigkeit wurden sowohl den Festangestellten ohne Nebentätigkeit als auch den Festangestellten mit Tätigkeit in und außerhalb der Leiharbeit gestellt. Eventuelle Gruppenunterschiede konnten für jede einzelne Frage mittels Varianzanalysen identifiziert werden (Abb. 3). Die Analyse ergab weder in Bezug auf die Zufriedenheit mit den Zukunftsaussichten, noch auf die Zufriedenheit in Bezug auf den Einsatz nach Fähigkeiten durch den Vorgesetzten signifikante Unterschiede der Gruppen Festangestellte ohne Nebenerwerb, Festangestellte mit Nebenerwerb und Festangestellte mit Nebenerwerb in der Leiharbeit. Die Gruppe der Festangestellten mit einem Nebenerwerb in Leiharbeit zeigte sich jedoch in den meisten untersuchten Variablen in puncto Zufriedenheit relativ gesehen als die unzufriedenste Gruppe (Tab. 4). Die Zukunftsaussichten werden, auf einer 5‑stufigen Sternskala, im Mittel mit einer 3 bewertet.

**Table 4:** 

Variable	F	*p*	$\nu_p^2$
Zukunftsaussichten Ihrer aktuellen Berufsausübung?	2,637	0,070	0,009
Art und Weise, in der Ihr direkter Vorgesetzter Ihre Fähigkeiten einsetzt?	1,533	0,204	0,006
Persönliche Entwicklungsmöglichkeiten?	3,064	0,027	0,012
Pflegedienstleitung insgesamt mit dem Personal umgeht?	3,629	0,013	0,014^a^
Arbeitgeber insgesamt mit den Mitarbeiter_innen umgeht?	1,700	0,166	0,007
Pflegedienstleitung mit Ihnen umgeht?	2,986	0,030	0,011^a^
Ärztlicher Dienst mit Ihnen umgeht?	0,052	0,984	0,000

^a^Signifkanter Post-hoc-Scheffé-Test für Festanstellung vs. Festanstellung und Nebenerwerb in Leiharbeit

### Wechselwilligkeit der Leiharbeiter_innen

Als initiale Gründe für den Austritt aus der Leiharbeit, bei denen eine Mehrfachantwort möglich war, gaben 128 (83 %) die höhere Vergütung (über dem Tarif) beim festen Arbeitgeber, 101 (66 %) einen festen Dienstplan nach eigenen Vorgaben, weitere 66 (43 %) die Zufriedenheit mit der aktuellen Tätigkeit an. Dabei gaben 47 (30 %) der TN an, auf Wechselschichten verzichten zu wollen. Wenn Leiharbeiter_innen sich für einen Schritt aus der Leiharbeit entscheiden würden, wären für 123 TN (78 %) eine zusätzliche Vergütung inkl. Freistellungen für weiterführende Tätigkeiten wie Praxisanleitung wichtig bis sehr wichtig. Trotz des festen Arbeitgebers finden 116 Befragte (75 %) es wichtig bis sehr wichtig, in unterschiedlichen Bereichen Berufserfahrung sammeln zu können (Tab. 5). Im Folgenden sollten die wichtigsten Gründe für einen Austritt aus der LA angegeben werden. Von diesen 142 TN gaben als wichtigsten Grund für den Austritt aus der Leiharbeit 66 (43 %) eine höhere Vergütung (über dem Tarif) an. Weitere 59 (39 %) gaben einen verlässlichen und festen Personalschlüssel inkl. feste und verlässliche Bettensperrungen bei Personalmangel an (Tab. 6).

**Table 5:** 

Grund	*n*	%
Höhere Vergütung als momentaner Tarif	128	83
Freistellung mit Zusatzvergütung (Praxisanleitung)	123	78
Feste Dienstpläne nach meinen Vorgaben	101	66
Zufriedenheit mit Tätigkeit	66	43
Karrieremöglichkeiten	62	40
Keine Wechselschicht	47	31
Dauerhafte Zugehörigkeit zu festem Team	44	29
Fester Dienstort	33	21
Persönliche Verbundenheit zum Arbeitgeber	32	21
Beteiligung an längerfristigen Aufgaben	31	20
Routine und Sicherheit beim Erfüllen der Aufgaben	27	18
Wechselnde Einsatzgebiete	23	15
Andere	17	11
Nicht wiederholt auftretende Einarbeitungsphase	17	11
Sicherheit des aktuellen Anstellungsverhältnis	14	9
Höherer Bezug zu Patient_innen	7	5

**Table 6:** 

Grund	*n*	%
Geld	66	43
Personal, fester Schlüssel	45	30
Verlässlicher Plan	14	9
Pausen	9	6
Wertschätzung	8	5

## Diskussion

Die Teilnehmer_innen dieser quantitativen Onlineumfrage unter 1203 TN aus dem Bereich der Fach- und Funktionspflege empfinden ein persönliches Nettoeinkommen von 3200 €/Monat als ausreichend und gerecht für ihre Tätigkeit auf deutschen Intensiv- und Intermediate-Care-Stationen. Die Leiharbeiter_innen in dieser Umfrage gaben dabei an, dass ein persönliches Nettoeinkommen von 3200 €/Monat ausreichend wäre, um von der Leiharbeit zurück in eine Festanstellung zu wechseln, sofern entsprechende Änderungen der Arbeitsbedingungen vorher umgesetzt wurden. Auch wurde festgestellt, dass der Wunsch, in die Leiharbeit zu gehen, geringer ausfällt, je höher das persönliche Nettoeinkommen ist.

Bisher wurde in vorherigen Umfragen kein genauer Wert zu den Gehaltswünschen abgefragt. Dabei ist nicht die Forderung nach mehr Gehalt der interessante Aspekt, vielmehr ist es der Umstand, dass die Pflegenden in dieser Umfrage unabhängig von soziökonomischen Ausgangswerten und unabhängig vom Arbeitsverhältnis eine im Median sehr ähnliche Angabe bei dieser Freitexteingabe zu den Gehaltsvorstellungen getätigt haben. Obwohl die Frage, in Bezug auf den bei dieser Frage anzunehmenden Stellenumfang für das angegebene Gehalt, nicht präzise genug gestellt war, ist durch alle Gruppen hinweg ein Gehalt von 3000 €/Monat netto als angemessen und gerecht bezeichnet worden und ab 3200 €/Monat netto sogar als ausreichend, um aus einer an vielen Stellen besser (> 3200 €/Monat) bezahlten Leiharbeit auszutreten.

Bemerkenswert ist, dass allein die Art der Anstellung und auch das höchste Gehalt nicht zufrieden machen, wenn die Arbeitsbedingungen als nicht gut empfunden werden. Dies spiegelt sich auch darin wider, dass die Gruppe der Festangestellten mit einem Nebenerwerb in Leiharbeit trotz des höheren persönlichen Nettoeinkommens in den meisten untersuchten Variablen die unzufriedenste Gruppe war.

Die Frage, was Pflegende aus einer Festanstellung im Detail bewegt in die Leiharbeit zu wechseln, kann auch mit dieser Arbeit nicht sicher abschließend beantwortet werden. Es gibt Hinweise darauf, dass es eine Typusfrage ist, sich für diesen Schritt zu entscheiden. Die NEXT-Studie von Simon et al. (2005) hat festgehalten, ohne einen Schwerpunkt auf die Intensivstation zu legen, dass als schlecht empfundene Arbeitsbedingungen und Vergütungen dazu führen, dass Pflegende ihren Beruf verlassen. Auch Karagiannidis et al. (2019) haben dies in ihrer Umfrage festgestellt, allerdings auch, dass 37 % zunächst von einer Reduzierung ihrer Stelle ausgehen. Gleichzeitig postuliert sogar der deutsche Gesetzgeber [3], dass in der Leiharbeit attraktivere Arbeitsbedingungen und höhere Löhne zum Abwerben genutzt werden. Es wäre also denkbar, dass ein Weg aus dem Beruf nicht nur eine reine „Alles-oder-Nichts“-Frage ist, sondern über den Weg einer Nebentätigkeit geschieht. Zumindest wäre das eine Erklärung dafür, warum die Festangestellten mit Nebenerwerb in der Leiharbeit unzufriedener sind als alle anderen Gruppen. Es kann als eine Art Karriereweg gesehen werden, dass die Unzufriedenheit in der Festanstellung erst über einen Nebenerwerb und mehr Gehalt kompensiert werden soll, um dann ganz aus der Patientenversorgung oder ganz in die Leiharbeit zu wechseln. Dies wird dadurch gestützt, dass in dieser Umfrage der Wunsch, in die Leiharbeit zu gehen, maßgeblich vom Einkommen abhing. Je höher das pNEK ist, desto geringer ist dieser Wunsch.

### Sichtweise auf die Arbeitsbedingungen

Den Wunsch, in die LA zu wechseln, rein auf die monetären Beweggründe zu beschränken, ist nicht gerechtfertigt. Zwar gaben die Leiharbeiter_innen dieser Umfrage eine höhere Vergütung als wichtigen Grund an, aus der Leiharbeit auszusteigen, allerdings bezogen sie sich lediglich auf den geltenden Tariflohn in der Festanstellung. Gleichwertig neben dem Gehalt wurde die Arbeitsplatzzufriedenheit genannt, die für oder gegen eine Tätigkeit in Festanstellung spricht.

### Stärken und Limitationen

Aufgrund der fehlenden systematischen Erfassung der quantitativen und qualitativen Besetzung von deutschen Intensivstationen können keine verlässlichen Rückschlüsse auf die Grundgesamtheit der Intensivpflegenden gezogen werden. Im Gegensatz hierzu wird die technische Ausstattung mit Betten und Beatmungsgeräten immer genauer überwacht [7]. Die Art der Verteilung des Fragebogens, insbesondere das Schneeballsystem, macht es unmöglich, eine Rücklaufquote zu berechnen.

Umfragen dieser Art sind mit einem Bias versehen. Es ist denkbar, dass sich nur bestimmte Personengruppen angesprochen fühlen, und ebenfalls, dass auch nur bestimmte Altersstrukturen angesprochen werden. Dabei kamen die Teilnehmer_innen mit einer kleinen Tendenz für NRW aus allen Bundesländern.

Angesichts einer großen Teilnehmer_innenzahl sind es jedoch über Einzelfälle hinausgehende Erkenntnisse, die gewonnen werden konnten. Der IP-Filter hat es nicht ermöglicht, mehrere Fragebögen vom selben PC auszufüllen. Dies beugt zum einem vor, dass Einzelpersonen mehrere Fragebögen ausfüllen, verhindert aber auch das mehrere Personen den gleichen z. B. Arbeitsrechner nutzen.

Um dem entgegenzuwirken, wurde in dieser Umfrage versucht, ein breites Spektrum verschiedener digitaler und analoger Medien sowie die persönliche Ansprache zu nutzen. Ferner bestehen bei einer Befragung mit Bewertungsskalen immer die Gefahr und die Möglichkeit einer Verzerrung [8, 22].

Um diesen Phänomenen zu begegnen, wurden die folgenden Punkte beachtet: Neben der Randomisierung der Fragen gab es ebenfalls eine sich abwechselnd ändernde Skalenorientierung in den modifizierten Likert-Skalen. Ebenso wurde nicht nach Zustimmung, sondern nach Meinung gefragt. Um eine Tendenz zur Mitte zu minimieren, wurde in der Skalierung das Item weiß nicht und unentschlossen weggelassen. Die Befragten mussten sich festlegen. Diese Punkte und die Möglichkeit, z. B. beim Gehalt eine Spanne oder Freitextantwort anzugeben, senken das Risiko für diese Verzerrungen. Einem Ankereffekt [24] wurde begegnet, indem z. B. bei den Gehaltsfragen die Frage nach dem persönlichen und Haushaltseinkommen an völlig anderer Stelle im Eingangsbogen gestellt wurde, als die Frage nach dem als gerecht empfundenen Gehalt.

Dass die Antworten, insbesondere beim Gehalt, als sozial erwünscht dargestellt wurden, ist in der Gesamtkonstellation sicherlich möglich, aber unwahrscheinlich. Das persönliche Gehalt passt zu den Angaben des Haushaltseinkommens und zeigt, dass Pflegende eher nicht die Hauptverdiener_innen sind. Unabhängig davon entspricht es der Lebenserfahrung, dass Festangestellte weniger verdienen als Festangestellte mit einem Nebenerwerb, was über alle Antworten signifikant herausgearbeitet wurde. Dass ein/e Leiharbeiter_in tendenziell mehr verdient als Festangestellte mit einem Nebenerwerb, deckt sich zumindest mit den Angaben der Bundesregierung in der Konzertierten Aktion Pflege.

### Schlussfolgerungen

Die Erkenntnisse dieser Umfrage bieten Hilfestellungen bei der als gerecht empfunden Gehaltsfrage und können neben der Ausgestaltung und Formulierung von guten Arbeitsbedingungen für Intensivpflegende helfen, Pflegende aus der Leiharbeit wieder in feste Teams zu rekrutieren und/oder in der Festanstellung zu halten und somit die qualitativ hochwertige Versorgung von Intensivpatient_innen zusätzlich sichern. Ferner könnten Maßnahmen zur Verbesserung der Arbeitsplatzbedingungen und monetäre Anreize formuliert werden, die gezielt Pflegekräfte in Leiharbeit motivieren, wieder in eine Festanstellung einer Klinik zu wechseln.

### Was muss sich ändern, um aus der Leiharbeit auszutreten?

Deckungsgleich mit den Äußerungen in dieser Umfrage, was sich ändern muss, um aus der Leiharbeit in die Festanstellung zurückzukehren, sind die von Isfort (2017) und Karagiannidis et al. (2019) als schlechte Arbeitsbedingungen identifizierten Umstände. Im Wesentlichen sind dies ein Fehlen von verlässlichen Dienstplänen, fehlende feste Personalschlüssel inkl. fester und verlässlicher Bettensperrungen bei Personalmangel, gefolgt von hohem Zeitdruck und ökonomischen Zwängen.

Trotz aller Bemühungen zur Verbesserung der Arbeitsbedingungen in den letzten Jahren fand die DGIIN [14] über eine Umfrage (*n* = 2498) heraus, dass 37 % der befragten Intensivpflegenden einen Ausstieg aus ihrem Beruf planen. Zudem wollen insgesamt 34 % der befragten Intensivpflegekräfte ihre Arbeitszeit in den nächsten 2 Jahren reduzieren [14]. Nicht wenige Pflegekräfte scheinen dabei aufgrund der aktuellen schlechten Arbeitsbedingungen in der Pflege momentan einen Wechsel in eine freiberufliche Tätigkeit bzw. in die Leiharbeit zu bevorzugen [2]. Dies ist insofern interessant, da die Befragten der DGIIN erst auf Platz 5 die bessere Bezahlung als Lösungsmöglichkeit angaben, während den folgenden Rahmenbedingungen eine höhere Bedeutung beigemessen wurde:

1. ein insgesamt besserer Personalschlüssel für die Stationen;
2. ein Betreuungsschlüssel von mindestens 1:2 in allen Schichten;
3. eine Verringerung der Arbeitsbelastung;
4. weniger Zeitdruck bei der Tätigkeit.

Welche Auswirkungen auf die Gesundheitsversorgung auf Intensiv- und IMC-Stationen durch die Leiharbeit auf Intensivstationen entstehen, lässt sich momentan nicht abschätzen. Insgesamt kann davon ausgegangen werden, dass die Qualität der Patient_innenversorgung mit der Gesamtqualifikation und Zusammensetzung des therapeutischen Teams zusammenhängt. Auch können auf einer Intensivstation, durch den insgesamt höheren Personalschlüssel gegenüber einer Normalstation, einzelne Qualitätsschwankungen in der Patient_innenversorgung durch das gesamte Team vermutlich besser aufgefangen werden.

Eine Auswirkung durch die Leiharbeit sind die finanziellen Effekte auf die jeweiligen Kliniken. Durch die Situation, dass auf eine hohe Nachfrage relativ wenig hochqualifizierte Pflegekräfte kommen, ist der Preis für diese Leistung im Verhältnis zu den Festangestellten deutlich höher. Der Umstand, dass verschiedene Vermittler und Agenturen zwischengeschaltet werden müssen, führt dazu, dass diese zwischengeschalteten Instanzen zusätzlich durch einen Preisaufschlag verdienen. Insgesamt ist festzuhalten, dass diejenigen, die sich für das Modell der Leiharbeit interessieren, sich dem auch hingezogen fühlen – unabhängig davon, wie die gesetzlichen Anforderungen sind. Sie werden sich diesem jeweiligen gesetzlichen Rahmen und den Situationen in der Regel anpassen. Wenn der Gesetzgeber der Leiharbeit effektiv begegnen möchte, ist ein Verbot nicht zielführend.

Vielmehr müssen die jeweiligen Verantwortlichen in den Kliniken neben diesen Arbeitsbedingungen vor Ort weitere Verbesserungen tätigen und sollten dabei auf die Bedarfe und Bedürfnisse ihrer Mitarbeiter_innen achten. Vor allem ein fester und verlässlicher Dienstplan und feste Betreuungsschlüssel sowie strukturierte Arbeitsbereiche mit einer im Dienstplan berücksichtigen Praxisanleitung scheinen hier der Schlüssel zum langjährigen Erfolg zu sein. Ein verlässlicher Dienstplan steht unmittelbar im Verhältnis zu einer ausgewogenen Work-Life-Balance.

Beim Einsatz von Leiharbeiter_innen ist dringend auf eine entsprechende Qualifikation zu achten. In dieser Umfrage ist deutlich geworden, dass viele Intensivpflegende eine lange Zugehörigkeit zu ihrem Beruf haben und wahrscheinlich durch diese enorme Berufserfahrung nicht unerheblich zur Kompensation von anderen Qualitätsschwankungen beitragen. Dies ist ein Umstand, auf den man sich in den nächsten Jahren nicht mehr verlassen kann, da immer mehr Pflegende entweder aktiv oder durch die Demographie bedingt aus ihrem Beruf ausscheiden werden. Die eigenen Qualitätsansprüche sollte die Berufsgruppe der Intensivpflegenden dabei idealerweise in einer Selbstverwaltung eigenständig bestimmen.

#### Kernaussagen

1. Intensivpflegenden dieser Umfrage empfinden aktuell im Median ein Gehalt von 3200 €/Monat netto als ausreichend und gerecht für ihre Tätigkeit.
2. Dieses Gehalt kann Leiharbeiter_innen bewegen zurück in die Festanstellung zu gehen.
3. Verbesserte Arbeitsbedingungen unabhängig vom Gehalt.
